# Targeting the endoplasmic reticulum with a membrane-interactive luminescent ruthenium(ii) polypyridyl complex†
†Electronic supplementary information (ESI) available: Experimental details, characterization of **2** and Fig. S1–S6. See DOI: 10.1039/c3sc51725j



**DOI:** 10.1039/c3sc51725j

**Published:** 2013-10-16

**Authors:** Martin R. Gill, Denis Cecchin, Michael G. Walker, Raminder S. Mulla, Giuseppe Battaglia, Carl Smythe, Jim A. Thomas

**Affiliations:** a Department of Chemistry , University of Sheffield , Sheffield , UK . Email: james.thomas@sheffield.ac.uk ; Fax: +44 (0)114 22 29436 ; Tel: +44 (0)114 22 29325; b Department of Biomedical Science , University of Sheffield , Sheffield , UK . Email: c.g.w.smythe@sheffield.ac.uk ; Fax: +44 (0)114 222 2787 ; Tel: +44 (0)114 222 2320; c Department of Chemistry , University College London , London , UK . Email: g.battaglia@ucl.ac.uk ; Tel: +44 (0)20 7679 4623

## Abstract

The characterization and bioactivity of the dinuclear ruthenium(ii) complex [(Ru(DIP)_2_)_2_(tpphz)]^4+^ (DIP = 4,7-diphenyl-1,10-phenanthroline and tpphz = tetrapyrido[3,2-*a*:2′,3′-*c*:3′′,2′′-*h*:2′′′,3′′′-*j*]phenazine) is reported. This new complex is found to be luminescent in acetonitrile, where excitation into MLCT (metal-to-ligand charge-transfer) bands in the visible area of the spectrum (*λ*_ex_ = 450 nm, *ε* = 45 000 M^–1^ cm^–1^) result in red emission (*λ*_em,max_ = 620 nm, *Φ*_MLCT_ = 0.017). Aqueous *in vitro* binding studies indicate that this complex binds to duplex DNA with an affinity of 1.8 × 10^6^ M^–1^ through a non-classical groove-binding interaction, however, unlike the parent complex [(Ru(phen)_2_)_2_(tpphz)]^4+^ (phen = 1,10-phenanthroline), it also displays an increase in MLCT luminescence on addition of liposomes. Confocal microscopy and TEM studies show that this lipophilic complex targets the endoplasmic reticulum of eukaryotic cells, where it functions as an imaging agent for this organelle, and cytotoxicity studies in human cancer cell lines indicate a comparable potency to the anti-cancer drug cisplatin.

## Abstract 2

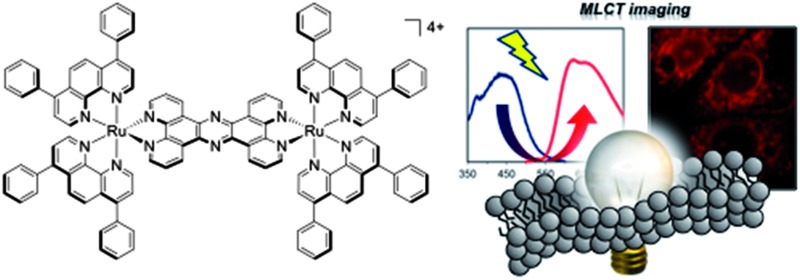
The characterization and bioactivity of the dinuclear ruthenium(ii) complex [(Ru(DIP)_2_)_2_(tpphz)]^4+^ (DIP = 4,7-diphenyl-1,10-phenanthroline and tpphz = tetrapyrido[3,2-*a*:2′,3′-*c*:3′′,2′′-*h*:2′′′,3′′′-*j*]phenazine) is reported.

## Introduction

Insight into how membrane-enclosed intracellular structures and organelles interact and are regulated within eukaryotic cells is of vital importance within cell biology and medicine.^1^ Arguably the two most notable organelles within the membrane-dense intracellular network are the endoplasmic reticulum (ER) and the Golgi apparatus. Apart from being involved in the assembly and transport of proteins, the ER also plays a key role in response to cellular stress, whilst the Golgi apparatus functions as an organisational centre for the cell.

The visualization of cellular structure by light microscopy remains a powerful research tool and, considering the number of commercially-available probes for DNA, there are very few small molecule analogues for ER and/or Golgi visualization. ER dyes include the lipophilic green-emissive DiOC_6_ (3,3′-dihexyloxacarbocyanine iodide) and the ER-tracker series, which consist of a boron-dipyrromethane (BODIPY) fluorophore conjugated to glibenclamide, a drug which targets ATP-sensitive K^+^ channels found in a high frequency on the ER.^2^ Disadvantages of these current commercial stains include small Stokes shift values (DiOC_6_ = 19 nm, ER Tracker Green = 7 nm, ER Tracker Red = 28 nm), and, in the case of DiOC_6_, additional mitochondrial targeting.

With the limitations of organic fluorophores in mind, there has been great interest in luminescent transition metal complexes as cellular imaging agents for optical microscopy.^3–5^ Ru^II^-based polypyridyl complexes are particularly attractive as their MLCT (metal-to-ligand charge-transfer)-based luminescence typically exhibit large Stokes shifts (*i.e.* >100 nm), accessible excitation energies in the visible (blue) region of the spectrum combined with red or far-red emission. These factors are advantageous as they mean that these complexes are compatible with existing experimental equipment, can be used as co-stains alongside established commercial fluorophores, and may negate problems resulting from cellular autofluorescence.

Since the discovery that [Ru(bpy)_2_(dppz)]^2+^ (bpy = 2,2′-bipyridine, dppz = dipyrido[3,2-*a*:2′,3′-*c*]phenazine) binds with high affinity to DNA and functions as a luminescence-based “light switch” (in which quenched luminescence in aqueous environment is activated upon non-covalent reversible DNA binding),^6^ a huge number of studies based on the interaction of octahedral ruthenium(ii) polypyridyl complexes with DNA have been published.^7,8^ In contrast, analogous *in vitro* interaction of Ru^II^ complexes with phospholipids and other self-assembling membrane structures has remained relatively unexplored. However, it is known that [Ru(bpy)_2_(dppz)]^2+^ itself functions as a luminescent indicator for sodium dodecyl sulfate micelle formation^9^ and a probe of lipid dynamics.^10^ More recently, the interaction of lipophillic Ru^II^dppz derivatives with lipid membranes has been explored.^11,12^ In these studies, it was found that the attachment of progressively longer alkyl chains to the dppz moiety resulted in a concomitant increase in membrane affinity.

**Scheme 1:**
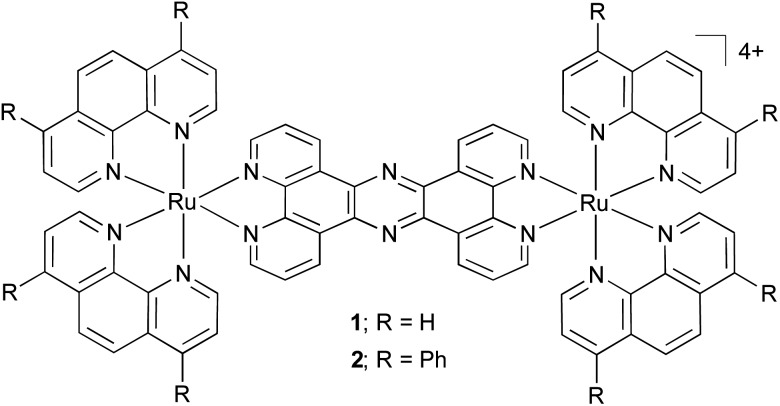
Dinuclear Ru^II^ complexes **1** and **2**.

In cellular studies, while a small number of luminescent Ru^II^ polypyridyl compounds capable of targeting nuclear DNA have been reported,^13^ studies into the cellular internalization of lipophilic luminescent Ru^II^ systems have frequently revealed non-nuclear localizations.^14–18^ While exact cellular targets have generally remained uncharacterized, membrane targeting has been strongly suggested in certain cases.^19–22^ This work clearly illustrates the potential for utilizing the photophysical properties of these complexes for the *in vitro* and *in cellulo* study of membrane structures.

Previously, we have reported on the cellular uptake properties of the dinuclear Ru^II^tpphz complex [(Ru(phen)_2_)_2_(tpphz)]^4+^ (phen = 1,10-phenanthroline, tpphz = tetrapyrido[3,2-*a*:2′,3′-*c*:3′′,2′′-*h*:2′′′,3′′′-*j*]phenazine), **1**, demonstrating this luminescent light switch complex functions as a DNA imaging agent (Scheme 1).^23^ In this study, we describe a lipophilic derivative of **1**, [(Ru(DIP)_2_)_2_(tpphz)]^4+^ (DIP = 4,7-diphenyl-1,10-phenanthroline), **2**, which is designed to localize within membrane structures; its *in vitro* interaction with both DNA and liposomes are reported and the potential of this complex for visualizing lipophilic intracellular structure by fluorescence (confocal) imaging and electron microscopy is assessed.

## Results and discussion

### Synthesis and characterisation of target complex

Previous studies have established that complex **1** targets the cell nucleus, where it binds to duplex DNA through reversible interactions.^23^ We reasoned that if the steric demands of this complex were greatly increased through larger ancillary ligands, then binding into duplex grooves would become increasingly unlikely. Aside from disfavouring DNA binding, the employment of more extended aromatic ancillary ligands would increase the lipophilicity of the final complex, thus favouring binding to lipid-based structures. Therefore, with these criteria in mind, the synthesis of complex **2** was carried out by adapting a previously reported method.^24^ The central tpphz bridging ligand was retained to facilitate the generation of an MLCT-active luminescent compound.^25^ The use of DIP as an ancillary ligand had the intended effect on lipophilicity: for **2** the estimated octanol–water partition coefficient, log *P*, is 1.52 ± 0.13, compared to –0.93 for **1**. An unavoidable drawback of this increase in the lipophilicity of **2** is that the complex displays relatively poor solubility in pure water, even as a chloride or nitrate salt. Accordingly, DMSO was employed to prepare working stock solutions of **2**, nevertheless the percentage of DMSO was kept to a maximum of 1% in the live cell uptake and cytotoxicity studies.

**Fig. 1:**
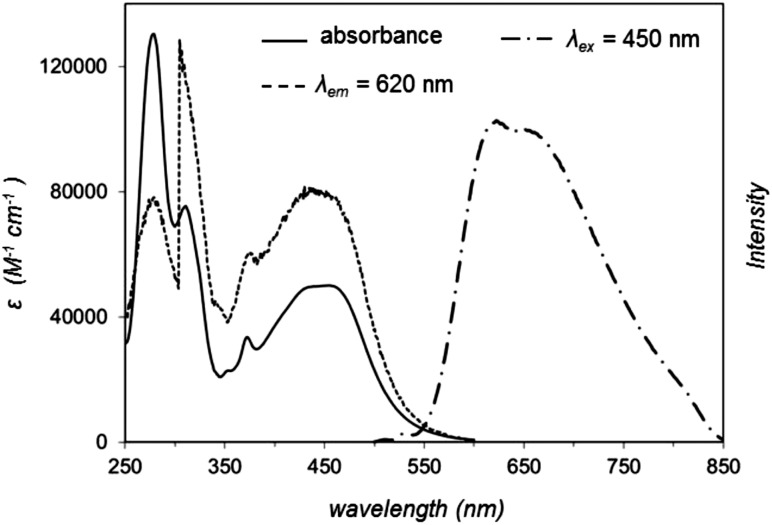
Absorption and emission (*λ*_ex_ = 450 nm) spectra of **2** in acetonitrile. The excitation spectrum (*λ*_em_ = 620 nm) is included for comparison.

The absorption and emission (*λ*_ex_ = 450 nm) spectra of **2** in acetonitrile are shown in Fig. 1. The electronic absorption spectrum for the complex displays high energy bands at 280 and 314 nm that are assigned to ligand-centred π → π* transitions, whilst the broad, low energy absorbances between 350 and 550 nm are characteristic of metal-to-ligand charge-transfer transitions from the Ru^II^ metal centres to the polypyridyl ligands. Complex **2** is luminescent in acetonitrile: ^1^MLCT excitation at 446 nm leads to ^3^MLCT-based emission centred at 620 nm, and the quantum yield for this emission is *Φ*_MLCT_ = 0.017 ± 0.002. This compares to a value of *Φ*_MLCT_ = 0.005 for **1** for an emission maxima centred at 690 nm.^26^ The increase in quantum yield and blue-shifted emission demonstrated by **2** are consistent with the energy gap law and suggest the ancillary ligands have a considerable effect on the MLCT excited state, consistent with our previous studies on Ru(dppz)-based systems incorporating extended polypyridyl ligands.^27^

### *In vitro* interaction with DNA or liposomes

**Fig. 2:**
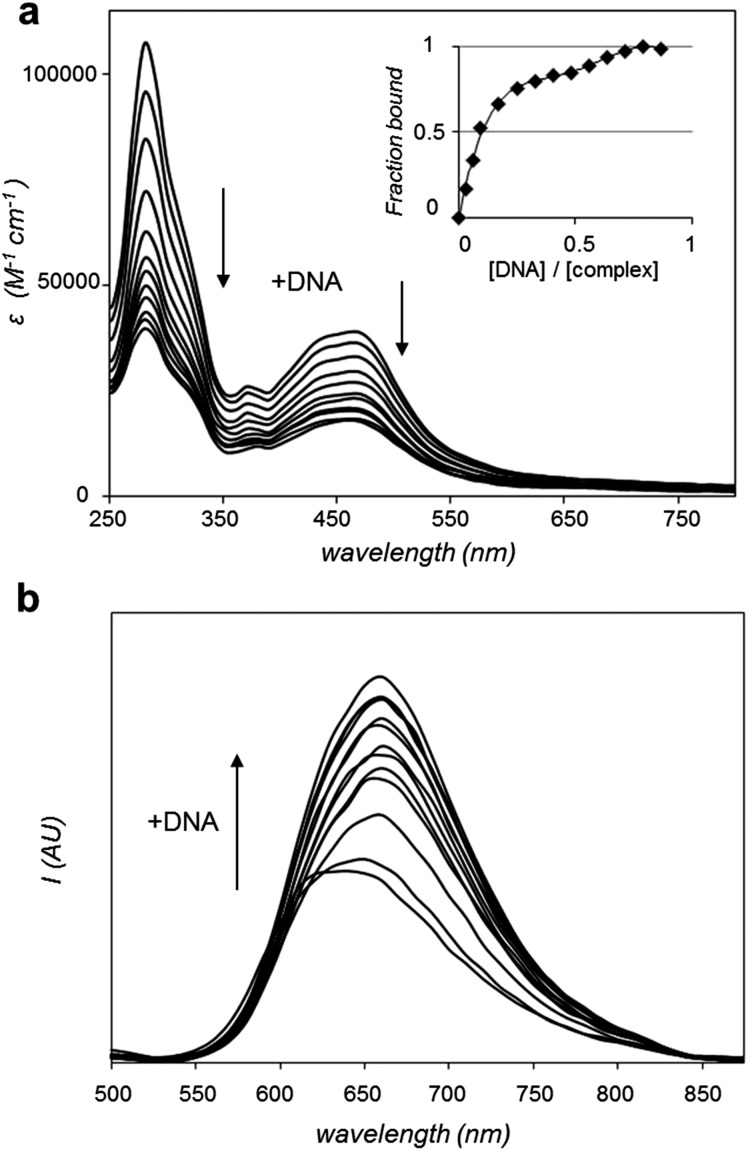
(a) Changes in UV-visible absorption spectrum of **2** (99% Tris buffer (5 mM tris(hydroxymethyl)aminomethane, 25 mM NaCl, pH 7), 1% DMSO) with addition of DNA, highlighting hypochromicity with increasing DNA concentration. Inset: derived binding curve from these data. (b) Increase in MLCT luminescence of **2** (*λ*_ex_ = 450 nm) with the addition of DNA.

Considering that **1** binds reversibly to duplex DNA with a high binding affinity,^28^ the ability of **2** to interact with DNA *in vitro* was investigated. Of particular interest was the question of how the extended aromatic ligand DIP would affect binding towards DNA in comparison to **1**. Changes in the UV-visible absorption spectrum of **2** upon the addition of increasing concentrations of calf thymus DNA resulted in appreciable hypochromicity for both π → π* and MLCT absorption bands of **2** (Fig. 2a). The changes in the π → π* band successfully yielded a saturation ligand–DNA binding curve (Fig. 2a, inset) and, by fitting these binding data to the McGhee von Hippel model,^29^ a derived binding constant, *K*_b_ = 1.8 × 10^6^ M^–1^ with a binding site size, *S* = 2.01 (in base pairs) was obtained. These results can be compared to *K*_b_ = 1.1 × 10^7^ M^–1^ and *S* = 2.6 previously reported for **1**,^28^ indicating **2** binds DNA an order of magnitude weaker than the parent complex. As can be seen in Fig. 2b, the interaction of **2** with DNA is accompanied by a two-fold enhancement of MLCT luminescence emission. This may be compared to a 60-fold increase demonstrated for **1**.^28^ Attempts to fit these small spectral changes to binding models failed to produce reliable saturation binding curves and thus a DNA binding constant could not be derived for **2** using this latter technique.

**Fig. 3:**
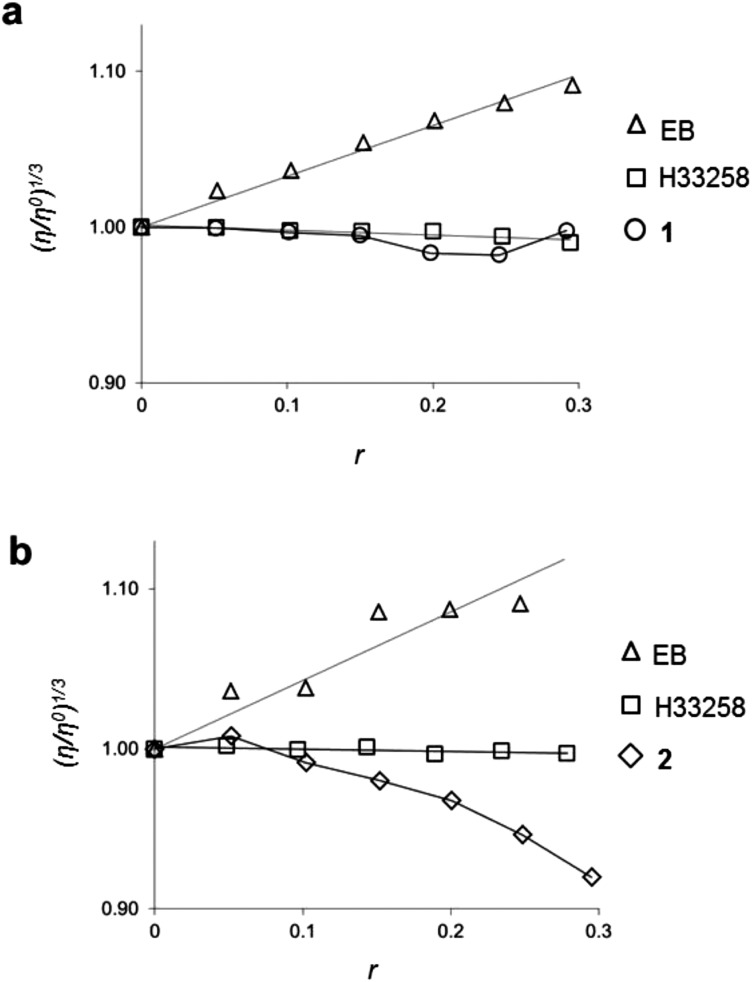
(a) Plot of relative viscosity (*η*/*η* ^0^)^1/3^ of DNA *versus r* (*r* = [complex]/[DNA]) upon addition of **1** (Tris buffer). (b) Plot of relative viscosity of DNA with addition of **2** (1% DMSO, 99% Tris buffer). The intercalator ethidium bromide (EB) and the groove binder Hoechst 33258 (H33258) were included for reference in both experimental conditions.

It is known^30–32^ that intercalating moieties unwind DNA, thus increasing relative viscosities of aqueous DNA solutions, while groove-binding molecules demonstrate no such effect. Therefore, to further explore the interaction of **2** and DNA, the relative viscosity of calf thymus DNA upon the addition of **2** was measured. **1** was included in these viscosity studies for comparative purposes. Fig. 3a indicates that the addition of **1** to DNA results in no change in the viscosity and, in contrast, the addition of **2** to a DNA solution actually results in a decrease of the specific viscosity (Fig. 3b). Taking Fig. 3a and b together, the behaviour of **2** clearly does not correlate with either an intercalating or classical groove binding mode of interaction with DNA. These results reveal that complex **1** is a groove-binding molecule in a similar manner to the bpy (2,2′-bipyridine) analogue, [(Ru(bpy)_2_)_2_(tpphz)]^4+^,^33^ and we conclude it is the greater steric bulk of **2** that inhibits the ability of this complex to associate with DNA through a canonical groove-binding interaction, resulting in a lower DNA binding affinity for the extended complex. Our viscosity studies indicate that complex **2** does distort the structure of DNA, and, in fact, the large diminution in relative viscosity observed indicates that **2** causes a decrease in the hydrodynamic length of DNA. A similar effect has previously been documented for Ru^II^ partial intercalators containing sterically hindering methyl groups^34^ as well as in our work on DNA binding metallomacrocycles.^35^ Considering that Fe^II^ helicates are able to induce intramolecular DNA coiling, with an accompanying decrease in DNA length,^36^ it is possible that **2** is similarly causing large scale bending of DNA. More detailed studies into the exact nature of DNA binding by **2** will form the basis of future work, however, in the remainder of this report we will focus on the interaction of **2** with bio-membranes.

**Fig. 4:**
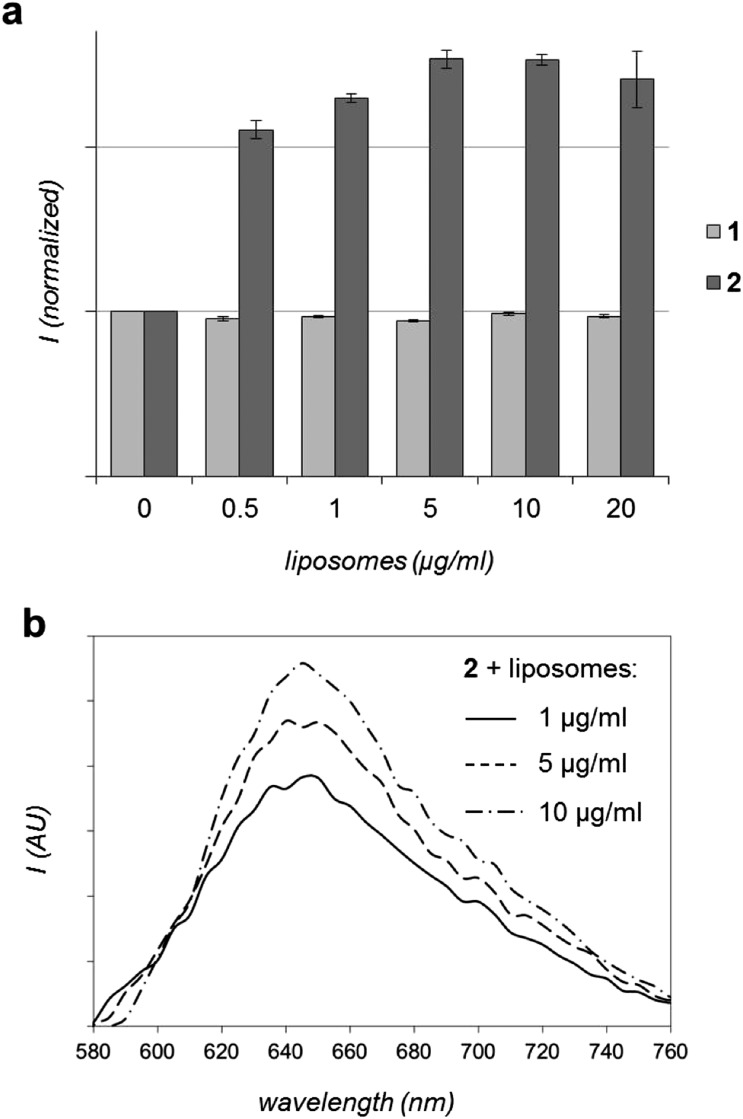
*In vitro* interaction of Ru^II^ complexes with liposomes. (a) Effect on MLCT luminescence intensity of **1** or **2** (*λ*_ex_ = 450 nm) with the addition of DOPC liposomes. Data presented as average of two readings ± SD. (b) Emission spectra of **2** (*λ*_ex_ = 488 nm) with the addition of DOPC liposomes (background corrected).

As discussed above, certain luminescent Ru^II^ polypyridyl compounds have an affinity for hydrophobic membranes. Since **2** is a hydrophobic molecule designed with this in mind, the *in vitro* interaction **2** with DOPC (DOPC = 1,2-dioleoyl-*sn-glycero*-3-phosphocholine, also known as dioleoylphosphatidylcholine) liposomes was then assessed. **1** was included as a hydrophilic MLCT luminescent control. As Fig. 4 shows, complex **2** demonstrates a clear increase in MLCT luminescence on addition of liposomes. Based upon the well-established photophysical properties of Ru^II^tpphz systems, it would seem highly likely that the interaction between **2** and liposomes results in increased shielding of the complex from water, which induces the observed increase in luminescence. In contrast, the addition of liposomes to **1** produces no changes in luminescence – even after extended incubation times. These contrasting responses indicate that the more lipophilic complex **2** interacts with liposomal membranes whereas no such interaction is observed for **1**.

Clearly – since the hydrophobic complex **2** displays an MLCT emission enhancement on association with liposomes – these *in vitro* experiments suggest that complex **2** has potential to target cellular membrane structures. This concept was addressed through detailed *in cellulo* studies.

### Cellular imaging studies

**Fig. 5:**
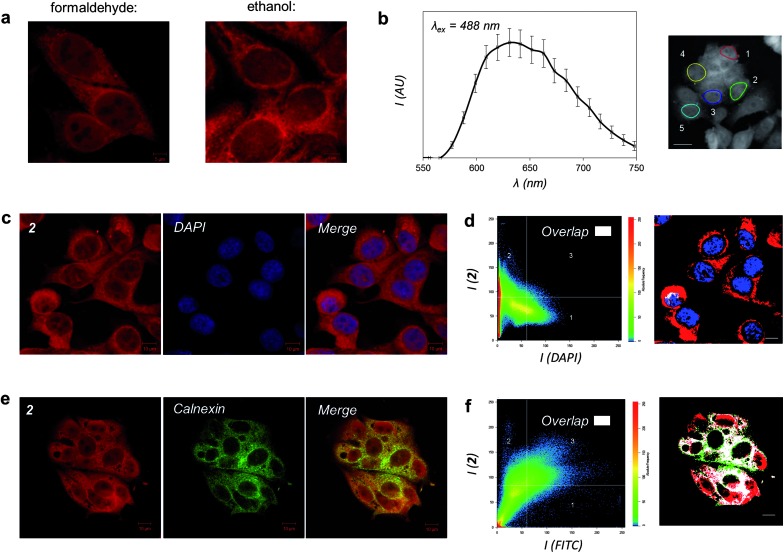
Cellular imaging properties of **2**. (a) MCF-7 cells stained by **2** after treatment with formaldehyde or ethanol fixation and visualised by MLCT excitation/emission wavelengths. (b) Emission profile of **2** in formaldehyde-fixed cells (*λ*_ex_ = 488 nm). Data presented as average emission of 5 regions of interest (right). (c) Co-staining with **2** and DNA dye DAPI. (d) Co-localization analysis of **2** and DAPI emission. Regions solely stained by **2** are labelled red, DAPI-exclusive regions blue, and regions of overlap of the two emission signals white. (e) Co-staining of cells stained with **2** and ER-localized calnexin protein, as visualized by immunofluorescence (FITC-conjugated secondary antibody). (f) Co-localization analysis of **2** and FITC emission where the region of overlapping signals is labelled in white. Scale bars in b, d, and f = 10 μm.

To facilitate detailed characterization of the imaging properties and cellular localization profile of **2**, MCF-7 human breast cancer cells were fixed utilizing two different methods before being exposed to solutions of the complex and visualized using fluorescence (confocal) microscopy. These studies particularly focused on the exciting possibility that this lipophilic Ru^II^ complex could behave as an MLCT imaging agent for lipid-dense cellular structures. As shown by representative micrographs in Fig. 5a, **2** is compatible with both formaldehyde and ethanol fixation. Fig. 5b and S1† demonstrate that **2** is compatible with laser excitation at either 458 or 488 nm both of which result in *in cellulo* emission centred at 640 nm, data in agreement with our *in vitro* studies outlined above. As 488 nm produces the optimal emission response in these microscopy conditions, this corresponds to a *de facto* Stokes shift value of 152 nm for the cellular MLCT luminescence of **2**. A detailed examination of the cellular localization profile of **2** following either fixation method demonstrates that **2** facilitates the visualization of specific internal cellular structures – of particular interest is the clear definition of nuclear membrane and/or perinuclear space.

To further explore intracellular cellular targeting, detailed co-localization experiments in which the overlap of emissions with established cellular fluorescent staining techniques were carried out. First, MCF-7 cells stained with **2** were co-stained with the nuclear dye DAPI (4′,6-diamidino-2-phenylindole). Cellular co-localisation analysis of the two luminescence signals reveals poor overlap of **2** and DAPI, indicating that the Ru^II^ complex does not strongly co-localize with the DNA dye (Fig. 5c and d). **1** was employed as a positive control for cellular DNA targeting and the strong co-localization between DAPI emission and the MLCT of **1** is evident (Fig. S2†). Combined with the observation that aggregated metaphase chromosomes remain unstained by **2** (Fig. S3†), this supplies compelling evidence that the lipophilic Ru^II^ complex **2** does not target cellular DNA. This behaviour is in agreement with our *in vitro* studies, and indicates that the sterically bulky DIP ligands of **2** inhibit strong *in cellulo* groove binding interactions with DNA.

As **2** is a lipophilic molecule which interacts strongly with membrane structures *in vitro*, we hypothesized that the molecule associates with lipophilic regions within the cell. Therefore, co-localization experiments for the ER and Golgi apparatus, two particularly lipid-dense organelles, were undertaken to fully establish the nature of the intracellular localization and emission of **2**. Immunofluorescent co-staining techniques were employed, whereby MCF-7 cells stained with **2** were probed using specific antibodies for proteins that localize in either the ER or Golgi apparatus; the cellular localization of each antibody then visualized using FITC (fluorescein isothiocyanate)-conjugated secondary antibodies. As FITC (*λ*_ex_ = 490 nm and *λ*_em_ = 525 nm; a Stokes shift of 35 nm) is likewise excited by 488 nm light, experiments to indicate that no bleed-through between the emission signals of FITC and **2** were first conducted (Fig. S4†). These experiments validated this methodology and, thanks to the significantly red-shifted emission wavelength of **2**, also validated the imaging compatibility of the complex with the commonly used FITC fluorophore. Immunofluorescent co-staining with **2** and calnexin, a calcium-binding protein located within the ER membrane, shows a clear overlap within MCF-7 cells (Fig. 5e). This is further confirmed by co-localization analysis of the two luminophores, showing a strong overlap of MLCT and FITC emission signals due to **2** and calnexin respectively (Fig. 5f). In contrast to these results, similar experiments employing immunofluorescent labelling with Golgin-97, a membrane protein located within the Golgi apparatus, demonstrated significantly poorer co-localization between FITC and **2** emission signals (Fig. S5†). Taken together, these results reveal that a significant proportion of **2** is ER-localized and the lipophilic Ru^II^ complex is targeting this organelle.

While ER-targeting has been established for luminescent europium(iii),^37,38^ zinc(ii)^39,40^ and platinum(ii)^41^ coordination complexes, and a range of metal-based systems have been postulated to demonstrate such behaviour,^21,22,42–46^ to the best of our knowledge this is the first time that a ruthenium(ii) complex has been definitively proven to be a luminescent cellular probe for this organelle. Furthermore, as shown in these studies, **2** demonstrates significant photophysical advantages over many commercial imaging agents: it has an easily accessible blue excitation, a large Stokes shift value of 152 nm and a red emission compatible with other existing fluorophores. Based on these data and our *in vitro* studies it is highly likely that the exact molecular target of **2** are lipid membranes and it is this interaction that enhances the Ru(tpphz)-based luminescence of the complex through shielding from water.

**Fig. 6:**
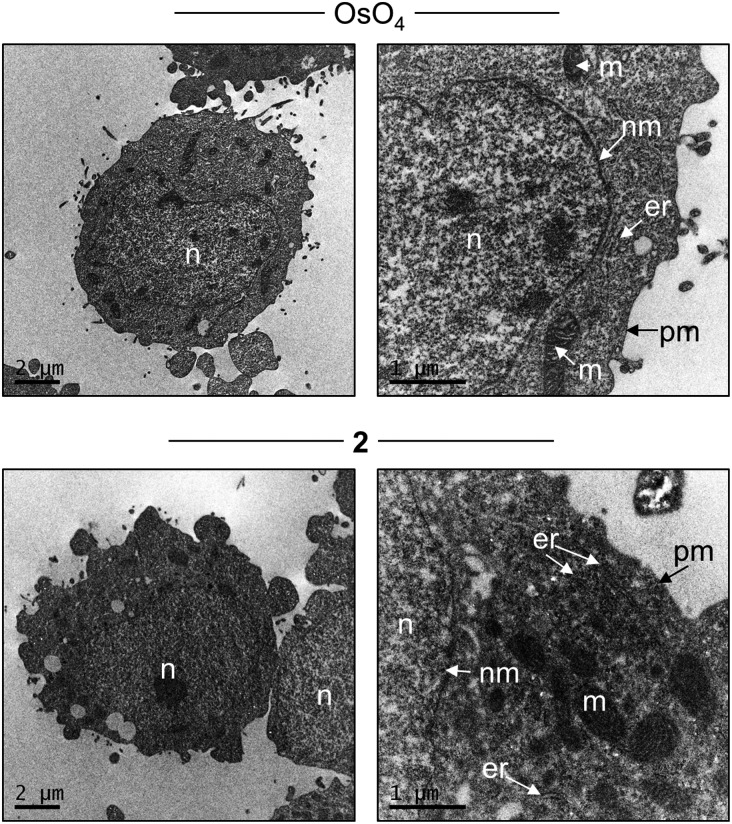
TEM of HeLa cells stained with either OsO_4_ (top) or **2** (bottom). Labelling: n = nucleus, nm = nuclear membrane, er = endoplasmic reticulum, m = mitochondria, pm = plasma membrane.

In addition to light microscopy, Ru^II^ polypyridyl compounds may function as contrast agents for transmission electron microscopy (TEM) analysis of cellular sections courtesy of the electron-dense Ru^II^ metal centre(s).^23,47,48^ With this in mind, the ability of **2** to function as a contrast stain for TEM was also explored. As shown in Fig. 6, staining fixed HeLa cells with **2** facilitates the visualization of internal cellular structure, where the addition of the Ru^II^ complex provides intracellular contrast levels comparable to cells stained with OsO_4_, a commonly employed TEM contrast agent that target lipids. Examining the intracellular localization of **2** at higher magnification, strong nuclear membrane and ER staining by **2** are evident while mitochondrial inner membrane structure is also defined using this high resolution technique (Fig. 6, btm right). Notably, **2** does not show a strong intranuclear contrast signal. These findings are in agreement with the light microscopy results in Fig. 5, and indicate that **2** has potential as a dual-mode cellular imaging agent for use with both light and electron microscopies.

### Live cell uptake and bioactivity

In addition to imaging studies, the biomedical application of luminescent metal complexes has been of growing interest.^49^ In such applications, the luminescent properties of Ru^II^ polypyridyl complexes may serve as tools to examine cellular uptake and targeting – two key factors in elucidating the mechanism of action of a potential therapeutic. Accordingly, the live cell uptake and impact upon cell viability of **2** towards two human cancer cell lines was investigated. We were particularly interested in exploring how a lipophilic, kinetically inert Ru^II^ compound that interacts with membranes would affect live cells.

To assess the cytotoxicity of **2**, MCF-7 human breast cancer and HeLa human cervical cancer cells were exposed to a concentration gradient of **2** and the resultant cell viability determined by MTT assay. From these data, half-inhibitory IC_50_ concentrations were extrapolated. The anti-cancer drug cisplatin was used as a positive control for the cytotoxic response of each cell line. As indicated by Table 1 and Fig. S6,† **2** was found to demonstrate appreciable cytotoxicity towards both cell lines, with IC_50_ values of 7 and 8 μM for MCF-7 and HeLa cells respectively; for MCF-7 cells this represents a 20-fold increase in cytotoxicity relative to **1** (IC_50_ = 138 μM). Examining the cellular morphology for each cell line exposed to cytotoxic levels of **2**, cell swelling (oncosis), cellular debris and evidence of intracellular vacuolization were apparent (Fig. S6†). As no indication of apoptotic cell morphologies (*e.g.* cell shrinkage/blebbing/fragmented nuclei) were observed, these changes are consistent with necrosis being the dominant cell death pathway, however, more detailed studies would be required to confirm this hypothesis.

**Table 1:** IC_50_ values (μM) of **1**, **2** and cisplatin towards MCF-7 and HeLa cancer cell lines (24 h). Data presented as average of three (**2**) or two (cisplatin) independent experiments ± SD

Complex	Cell line	
	MCF-7	HeLa
**1**	138 ± 6^*a*^	>200
**2**	7 ± 2	8 ± 1
Cisplatin	12 ± 3	24 ± 4

^*a*^Data from ref. 23.

**Fig. 7:**
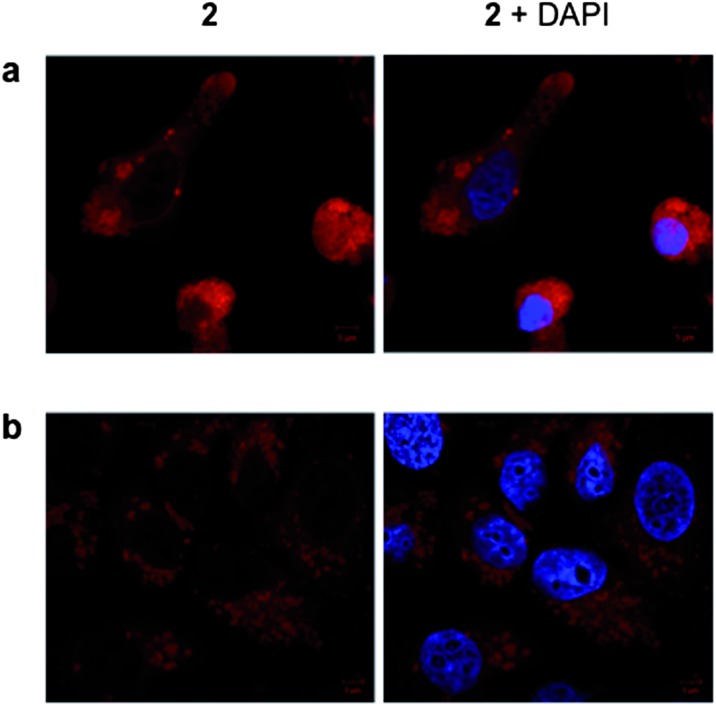
Live cell internalization of **2**. MCF-7 cells incubated with **2** (5 μM for 24 h) showing perinuclear (a) and intracellular vacuolar structures (b) stained by the Ru^II^ complex. Co-staining with DAPI is included (right hand column).

To examine the live cell uptake of **2**, MCF-7 cells were incubated with sub-IC_50_ concentrations of **2** before being visualized using confocal microscopy. As shown by Fig. 7, complex **2** is indeed internalized by live MCF-7 cells, where its cellular localization is observable by MLCT emission. Again, co-staining with DAPI confirms non-nuclear localization, and closer inspection reveals the compound is localized in the perinuclear region (Fig. 7a). This localization profile is in agreement with the fixed cells studies described above; furthermore, these live cell experiments also indicate that **2** is distributed in discrete vacuolar regions within the cytoplasm (Fig. 7b). Taken together, these results are consistent with targeting of the endomembrane system, which comprises of not just the ER but also the nuclear envelope and vacuole structures as well.

**Fig. 8:**
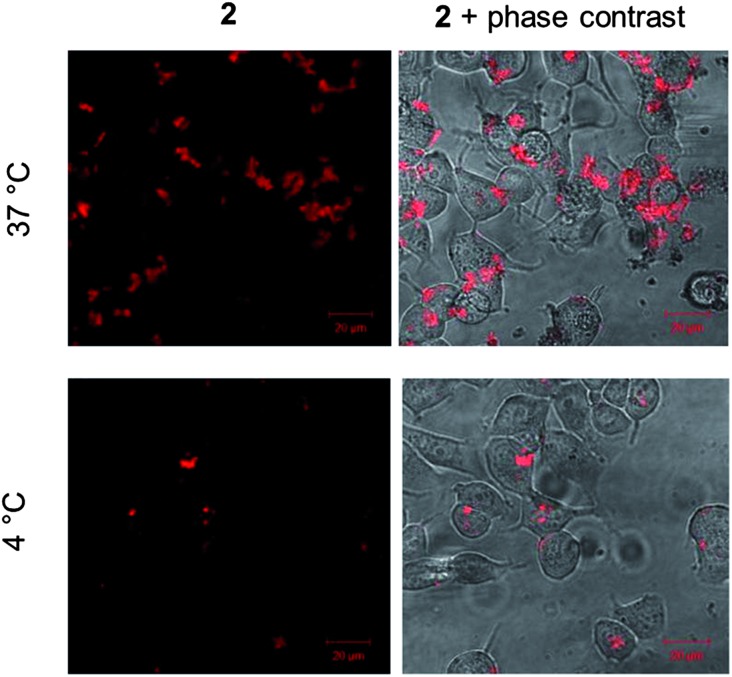
Temperature-dependent uptake of **2**. MCF-7 cells incubated with **2** (10 μM for 60 min) at either 37 °C (top) or 4 °C (bottom). Overlay of MLCT emission from **2** and phase contrast micrographs is included for reference (right hand column). Identical microscopy settings used for each incubation condition.

Given the live cell internalization of **2**, we then examined its cellular uptake mechanism, specifically to determine whether **2** is internalized by passive diffusion or by energy-dependent transport. To achieve this, MCF-7 cells were incubated with solutions of **2** at 37 °C or 4 °C, the latter conditions being employed to inhibit active uptake. The relative levels of cellular MLCT luminescence were then compared. As shown in Fig. 8, the cellular internalization of **2** is a temperature-dependent process, with cells incubated at 4 °C demonstrating significantly lower intracellular levels of **2** compared to cells incubated in parallel at 37 °C. This would indicate that a significant amount of cellular internalization of **2** is an active process although the low intracellular luminescence in cells incubated at 4 °C does suggest that a background level of passive diffusion also occurs.

Despite the lipophilicity of **2**, it is noteworthy that it is not internalized solely by passive diffusion. While work by Puckett and Barton demonstrated that increasing the lipophilicity of Ru^II^dppz complexes can promote cellular uptake by passive diffusion,^14,50^ this does not necessarily indicate passive diffusion is the dominant mechanism in all cases. Indeed, we have found active transport to be a common feature in our cellular uptake studies on mono- and dinuclear Ru^II^tpphz systems that are internalized rapidly by cells.^23,47^ Therefore, the results for **2** implying the involvement of an active transport uptake mechanism are consistent with this work. This emphasises the sensitivity to both lipophilicity and structure in the cellular uptake properties of Ru^II^ polypyridyl systems.

It should be pointed out that **2** was used as a racemic mixture within this study. Svensson *et al.* observed subtle differences in the fixed cell localization of DNA-targeting dinuclear Ru^II^ polypyridyl enantiomers, although no difference in live cell uptake was seen.^51^ It is not clear if this would similarly apply towards a membrane-interactive complex, however, it would be intriguing to examine both the spectral and bioactive properties of resolved stereoisomers of **2**.

Although restrictive in live cellular imaging applications, it is notable that **2** demonstrates significantly higher cytotoxicity than the DNA-targeting parent complex **1** towards two cancer cell lines. Largely due to the precedent set by platinum therapeutics, cytotoxic anti-cancer molecules that irreversibly target DNA have dominated inorganic medicinal chemistry,^52^ however, there has been growing interest in complexes that display affinity for other biological targets.^53^ For example, a recent report has described a reactive Ru^II^ organometallic anti-cancer compound that operates through the induction of ER stress.^54^ In this context, the fact that **2** has a comparable impact to cisplatin on human breast cancer cell viability and targets cellular membrane structures, including the ER, is significant in extending the scope of biological activities exhibited by kinetically inert octahedral metal complexes.

## Conclusions

In conclusion, we report the design and characterization of the lipophilic Ru^II^ MLCT luminescent complex **2**, which targets the lipid-dense endoplasmic reticulum in cells, where it acts as an *in cellulo* imaging agent for this organelle for confocal laser scanning microscopy and transmission electron microscopy. From the perspective of bioactivity, we observe a comparable cytotoxicity to the anti-cancer drug cisplatin, emphasising how control of the physical properties of this class of complexes can modulate cellular localization and, as a result, impact upon cell viability. Combined with our *in vitro* binding studies, we conclude the molecular target of **2** is biological membranes and future studies into the interaction of this complex with specific membrane structures will provide more detailed insights into the bioactivity of **2** and its analogues.
