# Supplementary material for: Targeting the endoplasmic reticulum with a membrane-interactive luminescent ruthenium(ii) polypyridyl complex
Source: Chem Sci. 2013 Oct 16;4(12):4512–9. doi: 10.1039/c3sc51725j (PMC4285105; doi:10.1039/c3sc51725j)
Supplement: Supplementary file 1 [file SC-004-C3SC51725J-s001.pdf]

Electronic Supplementary Material (ESI) for Chemical Science
This journal is © The Royal Society of Chemistry 2013

Targeting the endoplasmic reticulum with a membrane-interactive luminescent ruthenium(II) polypyridyl complex

Martin R. Gill,[a,b] Denis Cecchin,[c] Michael G. Walker,[a] Raminder S. Mulla,[a] Giuseppe Battaglia,*[c] Carl G. W. Smythe*[b] and Jim A. Thomas*[a]

[a] Department of Chemistry, University of Sheffield, Sheffield, UK. Fax: +44(0)114 22 29436; Tel: +44(0)114 22 29325; E-mail: james.thomas@sheffield.ac.uk [b] Department of Biomedical Science, University of Sheffield, Sheffield, UK. Fax: +44 (0)114 222 2787Tel: +44 (0)114 222 2320. E-mail: c.g.w.smythe@sheffield.ac.uk [c] Department of Chemistry, University College London, London, UK. Tel: +44 (0)20 7679 4623; E-mail: g.battaglia@ucl.ac.uk

Supplementary Information

Experimental section

All chemicals were purchased from Sigma Aldrich unless otherwise indicated.

Synthesis and characterization

1 was prepared from a synthetic method described previously.[1] **2** was synthesised by an adapted method of this, with DIP replacing phen in the experimental procedure. This involved the preparation of $Ru(DIP)_2Cl_2$ by a previously established pathway[2] before reaction with tpphz in a 1:1 solution of EtOH:water under reflux for 12 hrs. The addition of NH_4PF_6 caused the formation of a red/brown precipitate which was collected by filtration and washed with water. The crude product was recrystallised from acetonitrile/ether and dried *in vacuo*. Unless stated otherwise, **2** was used as the nitrate (NO_3^-) salt, which was obtained by anion metathesis. Mass (as PF_6^- salt) = 1.62 g (0.65 mmol, 73.8 %) red solid. MS; m/z (%): 1103 (55%) $[M-2(PF_6)]^{2+}$, 686 (100%) $[M-3(PF_6)]^{3+}$. ^{1}H NMR (400 MHz, MeCN-d^6): δ = 7.63 (d, J = 1.12 Hz, integration = 16H), 7.67 (s, 8H), 7.71 (d, J = 5.48 Hz, 4H), 8.03 (dd, J = 8.26, 5.42 Hz, 4H), 8.26 (d, J = 0.96 Hz, 16H), 8.33 (d, J = 5.52 Hz, 4H), 8.40 (dd, J = 5.48, 2 .00 Hz, 8H), 8.48 (dd, J = 5.38, 1.18 Hz, 8H), 10.06 (dd, J = 8.26, 1.18 Hz, 8H). Elemental analysis for **2**$(NO_3)_4.10H_2O$, $C_{120}H_{96}N_{18}O_{22}Ru_2$: Calcd: C; 61.48, H; 4.12, N; 10.75. Found: C; 61.83, H; 4.36, N; 10.50.

Absorption spectra were collected using a Cary 500 Scan UV-vis-NIR Spectrophotomer working in double beam mode (spectral band width = 2 nm) at a scan speed of 600 nm/min and baseline corrected. The excitation and emission spectrum was recorded using a Fluoromax-3 Spectrophotometer (excitation and emission slit = 5 nm, scan speed = 100 nm/min).

The quantum yield for **2** was calculated using Equation 1, where Φ_x and Φ_{ref} are the quantum yields of the sample and reference respectively, A_x and A_{ref} the measured absorbance of the sample and reference at the excitation wavelength, I_x and I_{ref} the integrated emission intensities of the sample and η_x and η_{ref} are the refractive index of the solvent of the sample and reference respectively.

$[Ru(bpy)_3](PF_6)_2$ in acetonitrile ($\Phi = 0.062$)[3] was used as the reference sample. A graph of *A* vs. *I* was plotted for **2** and $[Ru(bpy)_3](PF_6)_2$ from at least five data points.

$$\varphi_x = \varphi_{ref}\left(\frac{A_{ref}}{I_{ref}}\right)\left(\frac{I_x}{A_x}\right)\left(\frac{\eta_x}{\eta_{ref}}\right)^2$$

Equation 1 Quantum yield calculation

Estimated octanol/water partition coefficients for **2** were obtained using the "shake-flask" method, with the concentration in the octanol phase determined by UV-vis absorbance (Jasco V-630 Spectrophotometer). The partition coefficient, P, was calculated using $P = c_{oct}/ c_{H2O}$ (where c = concentration, and cH_2O calculated by *initial* c_{oct} – *final(measured)* c_{oct}).

In vitro interaction of 2 with DNA

Calf thymus DNA was dissolved in aqueous Tris-buffer (25 mM NaCl, 5 mM Tris, pH = 7) and broken into an average of 150-200 base pair fragments by sonication (2 × 15 mins). The purity of the sample was determined by UV-vis spectroscopy, with $A_{260}/A_{280} > 1.9$ indicating a protein-free sample. The concentration of DNA was determined by UV-vis spectroscopy, where $\varepsilon_{260nm} = 13200\ M^{-1}\ cm^{-1}$ (in bp). Luminescent titrations were carried out with the addition of a 118 µM aqueous solution of DNA to 2.5 µM **2**Cl_4 (1% DMSO, 99% Tris-buffer) in 2, 5, 10 and finally 15 µL doses. Between each addition, mixing was assured via the mechanical agitation of the fluorescence cell and employment of an equilibration period of 15 minutes. Emission spectra were taken at 25°C on a HORIBA Jobin Yvon Fluoromax 3 spectrofluorometer (operating at 5nm emission and excitation slit widths). For the UV-visible absorption titrations, a similar protocol was employed, where absorption spectra were recorded using a Cary 500 Scan UV-vis-NIR Spectrophotomer working in double beam mode (spectral band width = 2 nm) at a scan speed of 600 nm/min and baseline corrected. Absorption (hypochromicity) data were used to construct nonlinear Scatchard plots (r/Cf versus r) and fitted to the McGhee-von Hippel model[4] ($R^2 = 0.912$) in which neither the site size nor binding constant were defined. Viscosity experiments were performed as described in a recent publication.[5] Briefly, a 1 mL Cannon-Manning semi-micro viscometer (size 50) was maintained at 27 °C by immersion in a thermostated water bath. Additions of the ligand to ≈ 50 µM/ bp DNA were made so that the values of r (r = [ligand]/[DNA]) were between 0 and 0.3. An equilibration time of 20 min was allowed before the flow times were recorded. Times were recorded in triplicate to within 0.1 second of each other and averaged values obtained.

In vitro interaction of 2 with liposomes

DOPC (Avanti lipids) was solubilised in $CHCl_3$ and leaved to evaporate in a vacuum oven at 37 degrees for 2 hrs. The film was then re-hydrated with PBS pH 7.4 to a concentration of 50 mM. This was followed by 3 cycles of annealing at 60 degrees, followed by vortex. Each of the passages described were done for 3 minutes. This procedure allows a heterogenous suspension of liposomes to be obtained, which was left overnight at 4 °C. The homogenised liposome suspension was obtained by sonicating for 5 minutes before extrusion through a 0.22 µm filter membrane. Solutions of **1** or **2**

(5 μM, 0.1% DMSO: 99.9 % PBS) were then incubated with liposomes (0.5 – 20 μg/ml in PBS) in 96 well plates and allowed to equilibrate for 30 mins. The emission spectrum of each solution at 1, 6 and 24 hr timepoints was recorded by plate reader coupled to a Fluoromax 3 spectrofluorometer.

Cell culture and microscopy

MCF-7 human breast cancer cells were cultured in RPMI1640 medium (Invitrogen) supplemented with 10% fetal bovine serum (FBS) and penicillin/streptomycin. HeLa human cervical cancer cells were cultured in DMEM (Sigma) supplemented with 10% fetal bovine serum (FBS) and penicillin/streptomycin. For cell viability (cytotoxicity) assessment, cell cultures were treated with solutions of **1** (0.1 to 200 μM), **2** (0.01 μM to 10 μM) or cisplatin (0.01 μM to 100 μM) for 24 hours (in triplicate). Cell viability was quantified using 0.5 mg ml^{-1} MTT (MTT = (3-(4,5-Dimethylthiazol-2-yl)-2,5-diphenyltetrazolium bromide)) in serum-free media for 30 minutes. The formazan product was eluted using acidified isopropanol, absorbance at 540 nm quantified by spectrophotometer and cell viability determined as % untreated drug-free control. The IC_{50} value (the concentration that induces 50% viability) for each compound was calculated by interpolation from each corresponding Log C (M)/% viability plot. Images of cell cultures were obtained using a Dino-Lite AM423X Dino-Eye digital camera coupled to a Leica DMIL LED inverted microscope and processed using Dinocapture 2.0. For fixed cell imaging studies, cells were cultured on glass coverslips, fixed using either 10% formaldehyde or 70% ethanol and washed with PBS. Fixed cell samples were stained with 50 μM **2** (30% DMSO, 70% PBS) and washed with PBS. Samples additionally subjected to immunofluorescence staining were permeablized with 0.5% Triton X-100 in PBS for 10 mins, blocked in 5% bovine serum albumin (BSA) in PBS for 1 hr, and then immunostained with polyclonal antibodies that recognise either Calnexin or Golgin-97 temperature (antibodies courtesy of R. Piggott, University of Sheffield) at 1:100 dilution in BSA/PBS for 1 hr at room temperature. After three washes with PBS, primary antibodies were visualised via incubation with a fluorescein isothiocynate (FITC)-conjugated rabbit anti-mouse secondary antibody (Jackson ImmunoResearch Laboratories) at 1:200 dilution in PBS/BSA for 1 hr. To assess live cell uptake, MCF-7 cells were grown on microdishes (Thistle Scientific) and incubated with **2** (1% DMSO, 99% media) at 10 μM for 1 hr or 5 μM for 24 hrs. Temperature-dependent uptake studies used cells that had been cooled at 4°C for 60 minutes before incubation with **2**. Where stated, nuclear staining was performed using DAPI (500 nM in PBS) for 5 mins.

Cell cultures were luminescently imaged on a Zeiss LSM 510 META inverted confocal laser using 40x and 63x objectives. Ru(II) complexes **1** and **2** were excited with an Ar-ion laser at 458 nm or 488 nm and emission collected with a 575 nm (red) LP (long pass) filter. DAPI was excited using a 405 nm diode laser and emission detected using a 420-480 nm (blue) BP (band pass) filter. FITC-conjugated secondary antibodies were visualized using 488 nm Ar-ion excitation and a 505-550 nm (green) BP filter. Image data acquisition and processing was performed using Zeiss LSM Image software and co-localisation was performed using the Zeiss LSM image histogram analysis feature.

For TEM, HeLa cells were fixed using 3% glutaraldehyde. For OsO_4-stained cells, 2 % aqueous OsO_4 was applied for 2 hrs. Samples were dehydrated using a series of ethanol washes (70 – 100 % ethanol) and, where relevant, staining with **2** (2 mM in 100% ethanol) was applied at this point and samples left overnight. No other contrast stain than stated was applied at any point. Samples were sectioned in Araldite resin by microtome. Sections were examined on a FEI Tecnai instrument operating at 80 kV equipped with a Gatan 1 k CCD Camera.

Electronic Supplementary Material (ESI) for Chemical Science
This journal is © The Royal Society of Chemistry 2013

Supplementary Figures

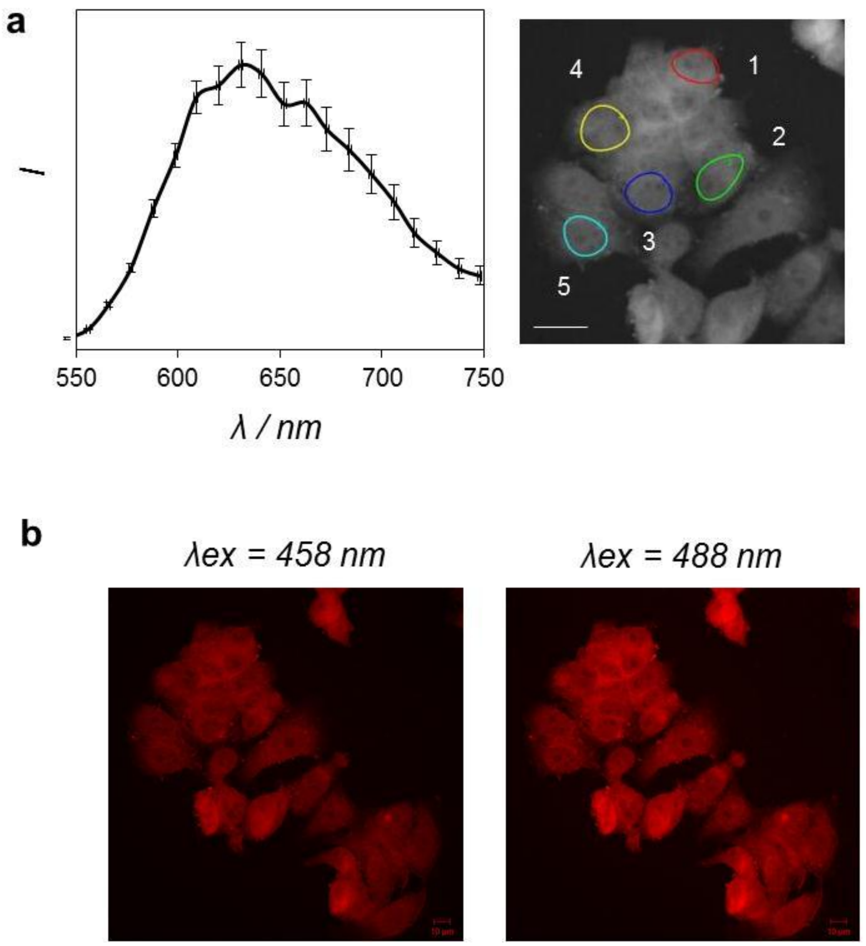

Supplementary Figure 1 (a) Emission profile of **2** in formaldehyde-fixed MCF-7 cells (λ_{ex} = 458 nm). Data presented as average emission of 5 regions of interest (highlighted to right). Scale bar = 10 µm. (b) Micrographs of MCF-7 cells stained with **2** excited by either 458 nm (left) or 488 nm (right) laser lines.

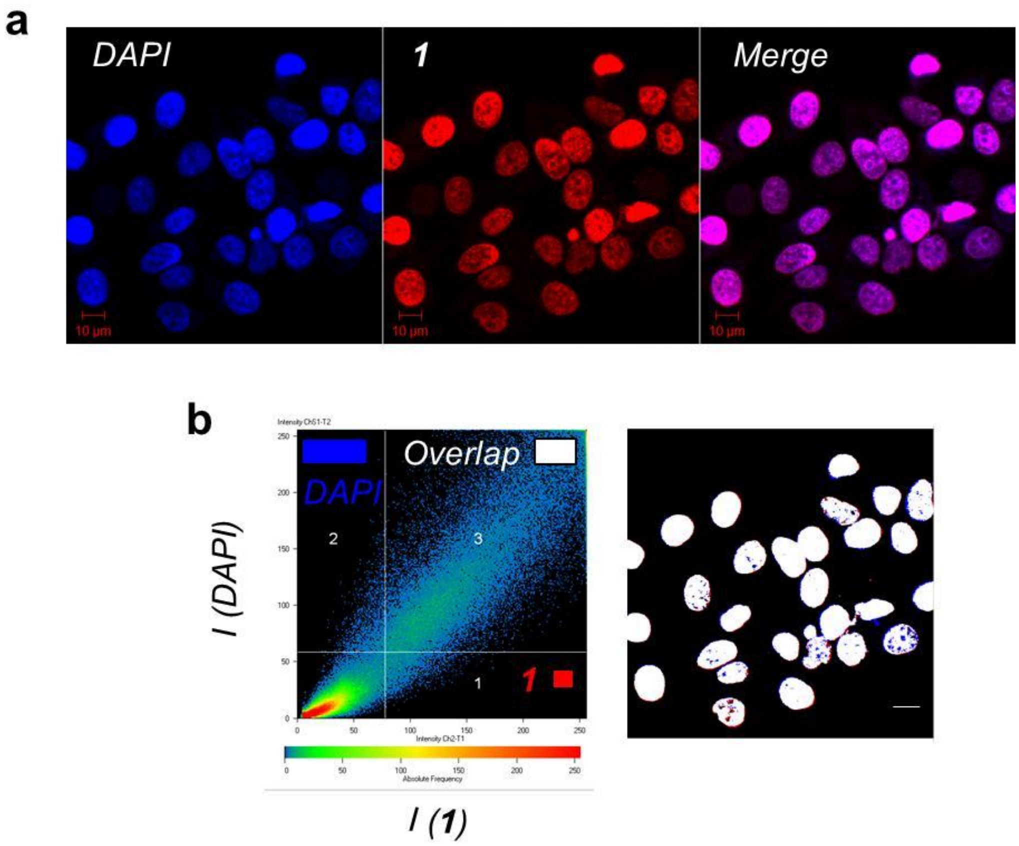

Supplementary Figure 2 (a) Co-staining of MCF-7 cells with **1** and DNA dye DAPI. (b) Co-localization analysis of **1** and DAPI emission. Regions solely stained by **1** and are labelled red, DAPI-exclusive regions blue, and regions of overlap of the two emission signals white.

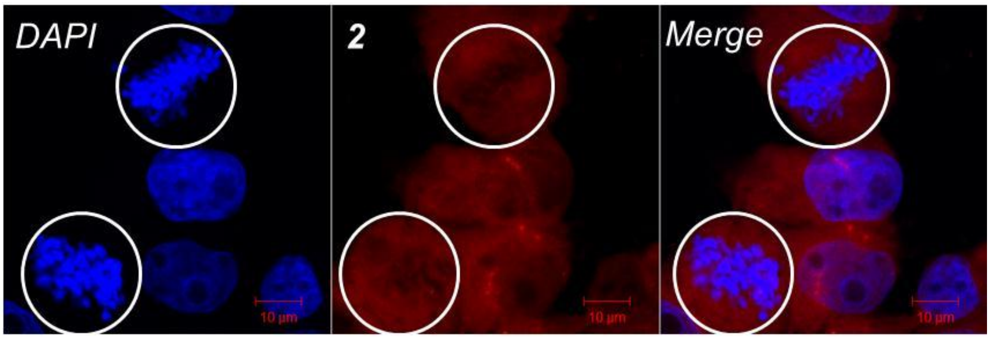

Supplementary Figure 3 MCF-7 metaphase chromosomes positively stained with DAPI (circled) remain unstained by **2**.

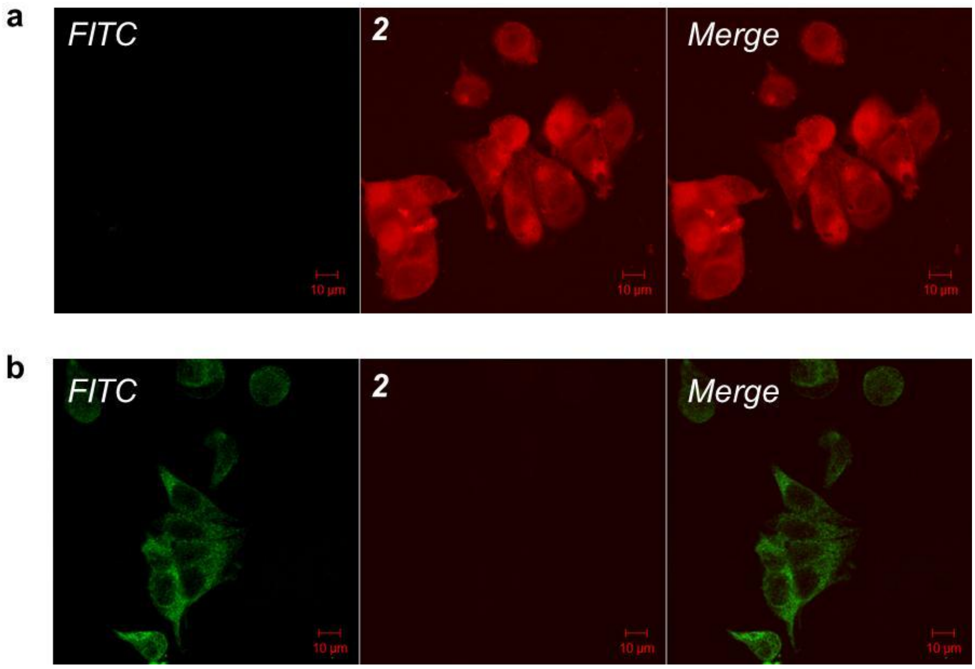

Supplementary Figure 4 Compatibility of **2** (MLCT) and FITC emission signals. (a) Formaldehyde-fixed MCF-7 cells stained with **2**. (b) Formaldehyde-fixed MCF-7 cells immunostained with Calnexin and FITC-conjugated secondary antibodies. These microscopy settings were used for all FITC/MLCT co-staining experiments.

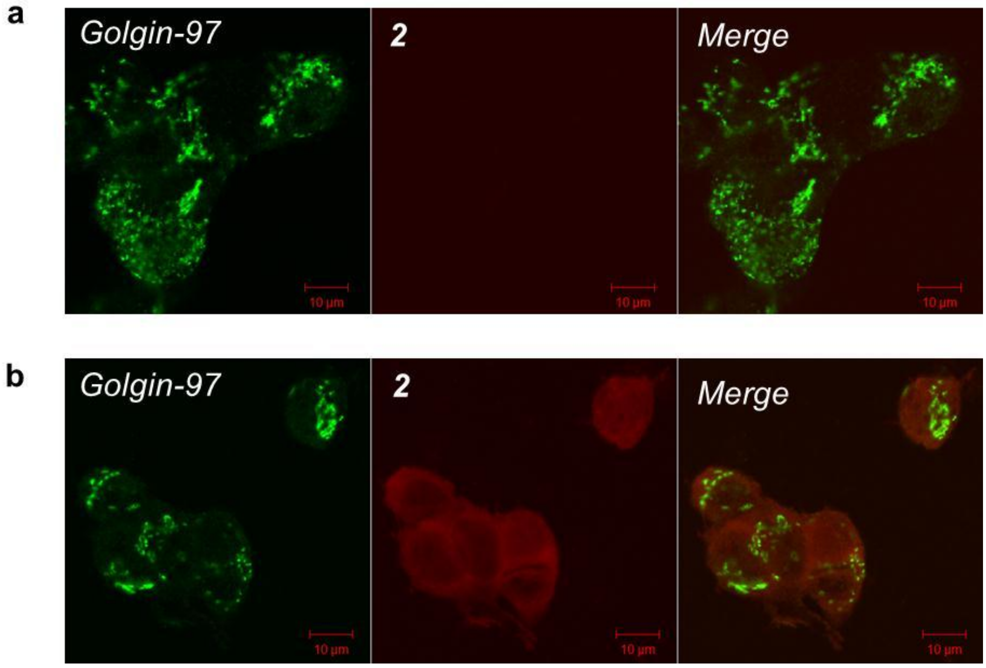

Supplementary Figure 5 (a) Formaldehyde-fixed MCF-7 cells immunostained with Golgin-97 and FITC-conjugated secondary antibodies. (b) Co-staining of MCF-7 cells stained with **2** and Golgin-97.

This journal is © The Royal Society of Chemistry 2013

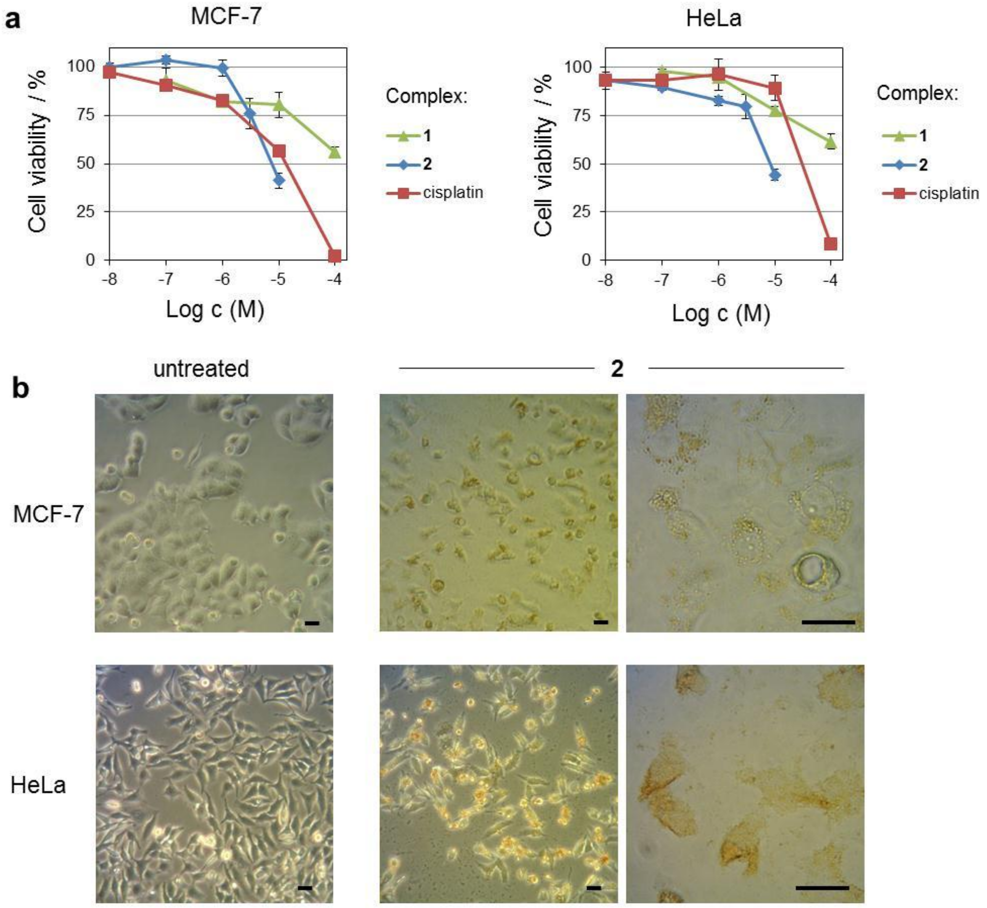

Supplementary Figure 6 (a) Effect of **1**, **2** or cisplatin on viability of MCF-7 (left) or HeLa (right) cells. Data presented as average of three (**2**) or two (**1**, cisplatin) independent experiments +/- SEM. (b) Representative images of MCF-7 (top) or HeLa (bottom) cells not-treated or treated with 10 µM **2**, highlighting the appearance of necrotic cells as a result of exposure to the Ru(II) complex (far right hand column). Scale bars = 20 µm.

References

1. J. Bolger, A. Gourdon, E. Ishow and J.-P. Launay, *Inorg. Chem.*, 1996, **35**, 2937.
2. B. P. Sullivan, D. J. Salmon and T. J. Meyer, *Inorg. Chem.*, 1978, **17**, 3334.
3. J. M. Calvert, J. V. Caspar, R. A. Binstead, T. D. Westmoreland and T. J. Meyer, *J. Am. Chem. Soc.*, 1982, **104**, 6620.
4. J. D. McGhee and P. H. von Hippel, *J. Mol. Biol.*, 1974, **86**, 469.
5. M. G. Walker, V. Gonzalez, E. Chekmeneva and J. A. Thomas, *Angew. Chem. Int. Ed.*, 2012, **51**, 12107.
